# Invasive Meningococcal Disease, Utah, 1995–2005

**DOI:** 10.3201/eid1308.061406

**Published:** 2007-08

**Authors:** Rachelle B. Boulton, Stephen C. Alder, Susan Mottice, A. Peter Catinella, Carrie L. Byington

**Affiliations:** * University of Utah, Salt Lake City, Utah, USA; † Utah Department of Health, Salt Lake City, Utah, USA

**Keywords:** Neisseria meningitidis, meningococcal infections, epidemiology, Utah, age distribution, Meningococci, serogroup B, serogroup Y, incidence, dispatch

## Abstract

Trends in invasive meningococcal disease in Utah during 1995–2005 have differed substantially from US trends in incidence rate and serogroup and age distributions. Regional surveillance is essential to identify high-risk populations that might benefit from targeted immunization efforts.

Invasive meningococcal disease (IMD) refers to the many illnesses caused by infection with *Neisseria meningitidis*. IMD is an immediately reportable disease in Utah and a nationally reportable disease in the United States. A preliminary review of IMD in Utah suggests that, since 2000, epidemiologic trends have occurred that are distinct from trends reported elsewhere in the United States. We describe the change in incidence rates, serogroup distribution, and age distribution of IMD in Utah, based on cases reported from 1995 through 2005, and compare our results with US trend data from the same period.

## The Study

We studied cases of IMD that occurred from January 1, 1995, through December 31, 2005, and were reported to the Utah Department of Health. Cases were classified as confirmed, probable, or suspected, based on the case definition for *N. meningitidis* infection in the Centers for Disease Control and Prevention and the Council of State and Territorial Epidemiologists 2005 case definition guidelines for IMD (*1*). Suspected cases, in which an isolate was not obtained, were not included in the final analysis because this study emphasized serotyping.

Utah incidence rates were calculated by using population estimates determined by Utah’s Indicator-Based Information System for Public Health (*2*). Incidence rates and serogroup distributions published in the *N. meningitidis* Active Bacterial Core surveillance (ABCs) reports were used to estimate US trends (*3*).

The Pearson χ^2^ test and Fisher exact test were used to test the statistical significance of the prevalence of serogroups by period for Utah and US data. Statistical analysis was performed with SAS (version 9.1; SAS Institute, Cary, NC, USA).

In the 10-year study period, 128 reported cases met the criteria of either confirmed or probable. Yearly incidence rates were calculated and ranged from a high of 0.95/100,000 population/year to a low of 0.21/100,000 population/year (Figure). Because the number of annual cases dropped after 1999, the data were divided into 2 periods. The mean incidence rate decreased significantly, by 63%, from 0.80/100,000 population/year from 1995 through 1999 (hereafter period 1) to 0.30/100,000 population/year from 2000 through 2005 (hereafter period 2).

**Figure:**
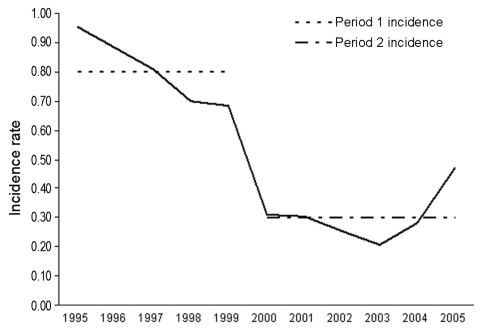
Incidence of invasive meningococcal disease by year, Utah, 1995–2005.

Incidence rates by period were stratified by age (Table 1). A rate difference was calculated by subtracting the average incidence rate for period 1 from the average incidence rate for period 2. The highest rate for both periods was for infants <1 year of age (period 1, average incidence rate 7.98/100,000 population/year; period 2, average incidence rate 3.07/100,000 population/year). The greatest rate difference also occurred for this age group, a decrease of 4.91/100,000 population/year between the 2 periods’ mean incidence rates (Table 1).

**Table 1:** Rates of invasive meningococcal disease by age group, Utah, 1995–2005*

Age, y	No. period 1 cases	No. period 2 cases	Period 1 rate/100,000	Period 2 rate/100,000	Rate difference
<1	17	9	7.98	3.07	−4.91
1–4	14	4	1.75	0.36	−1.39
5–14	10	4	0.53	0.17	−0.37
15–24	20	15	1.03	0.58	−0.45
25–34	4	3	0.25	0.26	0.01
35–44	6	2	0.40	0.11	−0.29
45–54	4	2	0.39	0.13	−0.26
55–64	3	2	0.48	0.20	−0.27
65–74	3	1	0.61	0.16	−0.46
75–84	2	1	0.66	0.24	−0.42
>85	1	1	1.03	0.68	−0.35
Total	84	44	0.80	0.30	−0.50

*Period 1, 1995–1999; period 2, 2000–2005.

The serogroup distribution in Utah changed substantially over the course of the 2 study periods. Before 2000, Utah meningococcal serogroup distribution reflected that of the United States; that is, serogroups B, C, and Y each caused ≈30% of IMD (*4*). Beginning in 2000, however, the percentage of serogroup B infections in Utah decreased significantly to 11.3%, while serogroup Y infections increased to 50.0% (p = 0.0102, Fisher exact test; χ^2^ = 7.2562, p = 0.0071). A similar change was not seen in US data. Whereas no significant difference was observed between Utah and ABCs data during period 1, a significant difference was seen for serogroups B (p = 0.0002) and Y (p < 0.0001) when period 2 data were compared (Table 2). Because of an ongoing outbreak of serogroup B disease in Oregon, Utah data were compared with US data both with and without Oregon’s numbers. For both comparisons, the conclusions were the same, and therefore Oregon’s numbers were not removed from the final analysis.

**Table 2:** Serogroup distribution of invasive meningococcal disease by period*

*Neisseria meningitidis* serogroup	Period 1				Period 2		
	Utah	United States	p value		Utah	United States	p value
B	32.1%	29.2%	0.5701		11.3%	39.7%	0.0002
Y	26.1%	32.0%	0.2773		50.0%	23.9%	<0.0001
C	25.0%	25.5%	0.9298		15.9%	21.4%	0.3783
Other	16.7%	13.4%	0.4084		22.7%	15.0%	0.1633

*Period 1, 1995–1999; period 2, 2000–2005. US data estimates based on information collected from Active Bacterial Core surveillance sites.

No Utah cases identified during the study period involved residents of military barracks or college dormitories, in which an increased risk for meningococcal disease is well documented (*5*,*6*). However, 5 (3.9%) patients were residents at a Job Corps facility (a residential job-training facility for young adults similar to a college dormitory). Of the 5 Job Corps cases, all were caused by serogroup Y infection, and 3 patients had bacteremic pneumonia.

The reduction in incidence rate could have several possible causes. One such cause could be a systematic change in reporting. However, no evidence to support this conclusion was found. Although the total number of reported cases declined between the 2 periods for most reporting hospitals, no single decline was strong enough to account for the observed decrease in reported cases. Underreporting of cases is another possible cause, but also is unlikely. Data from cases of IMD reported to the Utah Department of Health with onset dates from January 1, 2002, through December 31, 2005, were compared with data extracted from computerized laboratory records of a large hospital corporation in Utah for the same period. Ten cases of IMD were identified in each system, and demographic information confirmed that they were the same 10 patients.

Vaccination is unlikely to be the cause of the reduction in incidence rate as well. Over the study period, the percentage of vaccine-preventable strains causing disease in Utah increased, while infections caused by serogroup B, which is not included in the vaccine formula, decreased. Additionally, the greatest decrease in age-specific incidence rates occurred in age groups for which vaccination was not indicated.

Therefore, the decrease in the incidence rate seen is most likely the result of fluctuations in the community incidence rate, for which oscillations with a cyclical pattern have been documented (*7*–*9*). The incidence rate of IMD in Utah in 2005 increased substantially from the rate observed in 2004 (Figure). Although this rate is still much lower than rates seen for any year in period 1, it is still much greater than any other rate observed in period 2; the incidence rate appears to be increasing again, while the serogroup distribution is not changing. Due to the cyclical pattern of meningococcal disease, variability is expected, but the increase in serogroup Y cases and decrease in serogroup B cases appear unique to Utah.

## Conclusions

During the second study period (2000–2005), the incidence rate and age and serogroup distributions for IMD in Utah have differed from US trends. In Utah, the decrease in serogroup B infections, the most common cause of IMD in infants, resulted in an overall decrease in infections in infants and increased infection rates in adolescents and young adults ages 15 to 24 years. Furthermore, of the 38 serogrouped isolates from period 2, 31 (82%) were vaccine-preventable strains. This suggests that recommendations by the Advisory Committee on Immunization Practices (ACIP) for routine vaccination of selected cohorts with meningococcal conjugate vaccine (MCV-4) would be beneficial in Utah. ACIP recommendations, however, may not reflect regional epidemiologic trends. For example, Job Corp residents were identified as a high-risk population for IMD in Utah but have not been identified as a high-risk group in the United States. Because IMD is so rare, routine vaccination is costly (*10*), and vaccine supply is limited, we believe regional surveillance is a key factor in determining groups at high risk for IMD. The identification of serogroup Y disease among Job Corps residents influenced Utah’s vaccine policy. This study emphasizes the need for continued regional surveillance to help direct vaccine policy especially in regions of the United States not represented in ABCs.
